# Iranian 6-11 years age population-based EEG, ERP, and cognition dataset

**DOI:** 10.1038/s41597-025-04624-6

**Published:** 2025-02-22

**Authors:** Mohammad Ali Nazari, Sevda Abbasi, Maryam Rezaeian, Soomaayeh Heysieattalab, Hosein Safakheil, Ali Motie Nasrabadi, Zeynab Barzegar, Mohammad Taghi Joghataei, Zohreh Asgharian, Farshid Ghobadzadeh, Mohammadreza Alizadeh, Parvin Amini Yeganeh, Ayda Khayyat Naghadehi, Kiana Azizi, Mahdieh Alizadeh Chakharlou, AmirHossein Nasiri, Mohsen Davoudkhani, Mohsen Rezaeian, Mohsen Safakheil, Amirreza Katebi, Masoumeh Hasanzadeh Tahraband, Sahar Delkhahi, Haniyeh Soltani, Vajihe Shahrabi Farahani, Kimia Ghasemkhani, Erfan Nazari, Farhad Farkhondeh Tale Navi

**Affiliations:** 1 Department of Neuroscience, Faculty of Advanced Technologies in Medicine, Iran University of Medical Sciences (IUMS), Tehran, Iran; 2 Imâge Brain Institute, Tehran, Iran; 3 Department of Artificial Intelligence in Medicine, Faculty of Advanced Technologies in Medicine, Iran University of Medical Science, Tehran, Iran; 4 Department of Cognitive Neuroscience, Faculty of Education and Psychology, University of Tabriz, Tabriz, Iran; 5 Department of Biomedical Engineering, Faculty of Engineering, Shahed University, Tehran, Iran; 6 Cellular and Molecular Research Center, Iran University of Medical Sciences, Tehran, Iran; 7 School of Electrical and Computer Engineering, College of Engineering, University of Tehran, Tehran, Iran; 8 Department of Computer Science and Engineering, Shahid Beheshti University, Tehran, Iran; 9 Department of Counseling, Shahid Beheshti University, Tehran, Iran; 10 Department of Psychology, Faculty of Education and Psychology, University of Tabriz, Tabriz, Iran; 11 Department of Clinical Psychology, Faculty of Medical, Ahar Branch, Islamic Azad University, Ahar, Iran; 12 Department of Psychology, Karaj Branch, Islamic Azad University, Karaj, Iran; 13 Department of Psychological Sciences, Kansas State University, Kansas, USA; 14 Epidemiology and Biostatistics Department, School of Health, Occupational Environment Research Center, Rafsanjan University of Medical Sciences, Rafsanjan, Iran; 15 Department of Psychology, Faculty of Psychology and Educational Sciences, South Tehran Branch, Islamic Azad University, Tehran, Iran; 16 Department of Audiology, School of Rehabilitation, Shahid Beheshti University of Medical Sciences, Tehran, Iran; 17 Department of Psychology, University of Milano-Bicocca, Milan, Italy; 18 Institute for Cognitive Science Studies (ICSS), Tehran, Iran; 19 Department of Psychology, Science and Research Branch, Islamic Azad University, Rudehen, Iran; 20 Life Sciences and Biotechnology, Shahid Beheshti University, Tehran, Iran

**Keywords:** Paediatric research, Predictive markers

## Abstract

This report presents an open-source dataset investigating neurodevelopmental profiles in children. The dataset consists of EEG, ERP, and cognitive assessments from 100 Iranian non-clinical participants (age range 6–11 years, Mean = 8.52 ± 1.5 SD). Notably, this is a smaller group drawn from a larger longitudinal ongoing study. The research aligns with the Research Domain Criteria (RDoC) framework, aiming to enhance diagnostic precision and intervention efficacy for specific learning disabilities (SLD) using EEG/ERP measures and machine learning. Cognitive assessments included non-verbal intelligence (Raven Test), attention (IVA-2), and working memory tasks. EEG recordings captured resting-state (eyes closed/open) and brain activity during working memory tasks with numerical and non-numerical stimuli (ERPs). Additionally, demographic information such as age, gender, education, handedness, parental history of learning difficulties, and child symptom inventory-4 (CSI-4) were collected. This dataset provides a valuable resource for exploring the neurophysiological correlates of cognitive functions in typically developing children, which can advance our understanding of the neural foundations of cognitive development in children.

## Background & Summary

Children aged 6 to 12 undergo a crucial developmental stage that profoundly influences fundamental cognitive processes, affecting both their academic performance and overall mental well-being^1,2^. Challenges such as learning disabilities, attention and memory issues, and mood disorders can significantly impact various aspects of their lives during this period^3^. Recognizing the importance of this developmental stage, it is crucial to address and resolve these issues proactively^1^.

In accordance with the Diagnostic and Statistical Manual of Mental Disorders, fifth edition (DSM-5), specific learning disabilities (SLD) are characterized by unexpected difficulties persisting for at least six months in reading, writing, and mathematics. Dyslexia refers to a specific deficiency in word recognition and decoding, leading to reading impairments^4^. On the other hand, dyscalculia manifests as deficiencies in number fact knowledge and computation, impacting arithmetic skills^5^. The estimated prevalence of dyslexia and dyscalculia is approximately 5-12% and 3.5–6.5% among children, respectively^6^.

This study aligns with the Research Domain Criteria (RDoC) project, a National Institute of Mental Health (NIMH) initiative launched in 2008. RDoC aims to develop a more precise classification system for mental disorders by linking observable behaviors with underlying neural functions^7^. Departing from conventional classifications like DSM and International Classification of Diseases (ICD), RDoC seeks to improve diagnosis, treatment, and prognosis for psychiatric disorders by identifying the biological underpinnings of mental illness^8^.

The conventional DSM classification, relying on semiotics and behavioral symptoms, often leads to delayed diagnosis of SLD in children, missing the crucial window for effective interventions and hindering their learning and development^9^. Research emphasizes the importance of early intervention, as it is far more effective than addressing issues after academic difficulties arise^10^. The RDoC framework offers a novel approach by considering phenotypic diversity in speech, cognitive functions, and social functioning in these children^11^. This shift towards a biological understanding of SLD has the potential to improve diagnosis accuracy, enabling earlier intervention and leading to better learning outcomes.

Learning disabilities are associated with deficits in areas such as phonological processing, working memory, numerical magnitude processing, attention, and processing speed^12^. While studies have identified various brain dysfunctions in children with SLD, researchers are actively searching for valid neuromarkers (biological indicators) within their nervous systems. Event-Related Potentials (ERPs) offer advantages due to their high temporal resolution, providing a detailed map of brain information processing during cognitive tasks^13^. This high temporal resolution is important for understanding the precise timing of neural activity potentially disrupted in SLD.

This project employs a combined longitudinal and cross-sectional study design, focusing on children aged 6 and above with a two-year follow-up to observe developmental changes. Investigating neural correlates of working memory, attention, and emotional problems in children with SLD using EEG/ERP recordings, the study aims to identify markers distinguishing dyslexia, dyscalculia, and typical development. This early and accurate diagnosis will be crucial for implementing effective, targeted interventions for SLD. To achieve this objective, we are developing a machine learning-based classification system for SLD subtypes (dyslexia, dyscalculia) prediction using EEG/ERP and behavioral data. Ultimately, this research aspires to translate the findings into a practical protocol for early SLD detection in clinical settings, facilitating timely interventions and improved learning outcomes.

## Methods

### Participants

A subset of 100 participants (mean age = 8.52 ± 1.5 SD) was drawn from a larger, ongoing longitudinal study. Detailed demographic information, including gender and age distribution, is provided in Table 1. Data were collected from Tehran (and Karaj), and Tabriz cities of Iran.

**Table 1:** Demographic description of the sample.

	Categories		n = 100
Gender	Male		**45**
	Female		**55**
Age	6	Male	**12**
		Female	**13**
	7	Male	**11**
		Female	**7**
	8	Male	**7**
		Female	**7**
	9	Male	**9**
		Female	**8**
	10	Male	**12**
		Female	**7**
	11	Male	**4**
		Female	**2**

According to the primary goal of the project, children aged 6 were specifically recruited for the longitudinal study, while older children were included in the cross-sectional study. Special emphasis has been placed on enrolling an adequate number of participants within the 6-year-old age group to ensure robust data for the longitudinal study. The data collection for this segment of the study is expected to continue for approximately two years.

### Ethics

Prior to participation in the study, all participants’ parents or legal guardians received both verbal and written information about the research. Informed consent, designed under the supervision of the ethical committee, was voluntarily obtained from them. The informed consent process included detailed explanations about the study procedures, the devices used, safety precautions, and the data sharing policy. Ethical approval for data collection and sharing, while ensuring the preservation of participants’ personal information, was obtained from the regional ethics committees of the University of Tabriz (permit No: IR.TBZMED.RCE.1401.069) and Iran University of Medical Sciences (permit No: IR.IUMS.REC.1401.349) for Tabriz and Tehran (including Karaj), respectively.

### Exclusion criteria

Both longitudinal and cross-sectional participants were excluded if they had any medical conditions, neurological diseases, or psychiatric disorders. This criterion is particularly important for the follow-up study of the 6-year-old group, as it aims to identify children with potential learning disorders, which, according to DSM diagnostic criteria, must not be confounded by auditory, visual, motor, intellectual, neurological, or psychiatric disorders.

### Procedure

**Fig. 1:**
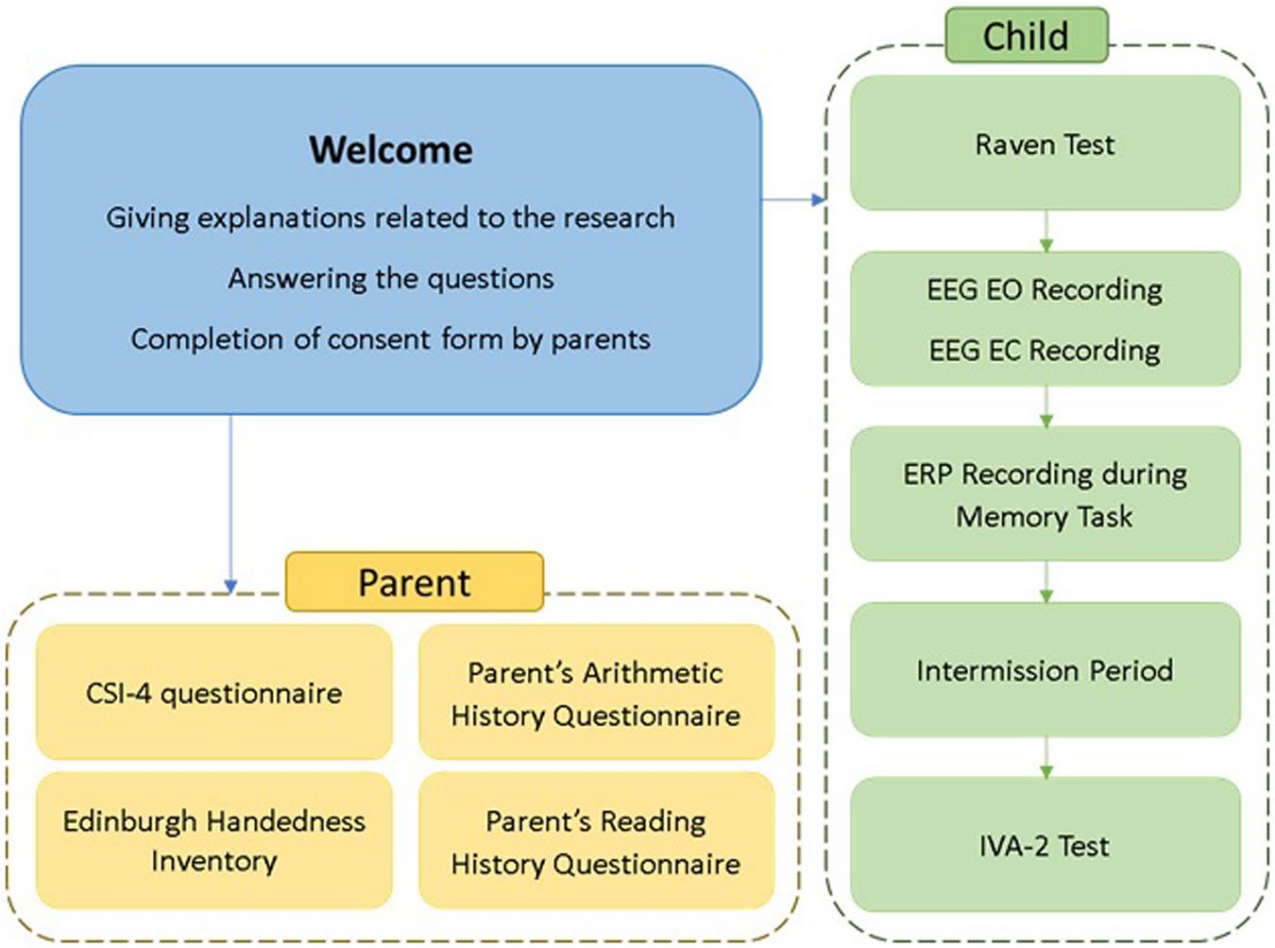
Flowchart of Procedure.

Participants were randomly selected from preschools and schools affiliated with the Ministry of Education. The sequence of the tasks conducted with participants is outlined in Fig. 1. Initially, each participant received a unique code for identification throughout the study. The Raven’s Progressive Matrices (commonly referred to as the Raven test) was administered in a child-friendly manner to establish rapport between children and the experimenter. This initial step typically lasted around 20 minutes. If the child showed no signs of fatigue, they were guided to a dedicated recording room for EEG assessment. Resting-states (rs) EEG recordings were conducted during two conditions: eyes open (EO) for 4 minutes and eyes closed (EC) for 4 minutes. Then, children were instructed to complete two working memory tasks for the recording of their ERPs. The order of the tasks was counterbalanced to mitigate any potential order effects.

Once the EEG/ERP recording was completed, the EEG cap was gently removed, and any residual gel was carefully cleaned from the child’s scalp. Each child was then thanked for their cooperation and offered a break lasting at least 30 minutes, with the option for a longer duration if needed. During this break, all participants had the opportunity to rest and refreshments were provided. Following the break, if the child showed no signs of fatigue and expressed the willingness to proceed, the IVA-2 test was conducted. Throughout these procedures, the parents of each child completed various questionnaires on a tablet, including the Child Symptom Inventory–4 (CSI–4), Edinburgh Handedness Inventory (EHI), Adult Reading History Questionnaire (ARHQ), and Adult Arithmetic History Questionnaire (AAHQ). To express our gratitude for their participation in the study, a comprehensive evaluation of each child’s cognitive and psychological state was offered to their parents. Our team addressed any questions the parents had regarding the assessment and results. All data were collected and analyzed using the NeurokidMap software developed by the Imâge Brain Institute.

## Cognitive Tasks

### Integrated visual and auditory continuous performance test, second edition (IVA-2)

The Continuous Performance Tests (CPT) is a common paradigm used by researchers and clinicians to assess sustained and selective attention. The IVA-2, a unique CPT, stands out by simultaneously testing auditory and visual performance. Normed on a diverse group of over 1,700 participants aged 6 and above, it has demonstrated high sensitivity (92%) and accuracy (90%) in detecting ADHD in children aged 7 to 12^14^. In a broader age range study (6 to 55), it matched clinical diagnoses 90% of the time and achieved 89% accuracy in identifying non-ADHD cases^15^.

**Fig. 2:**
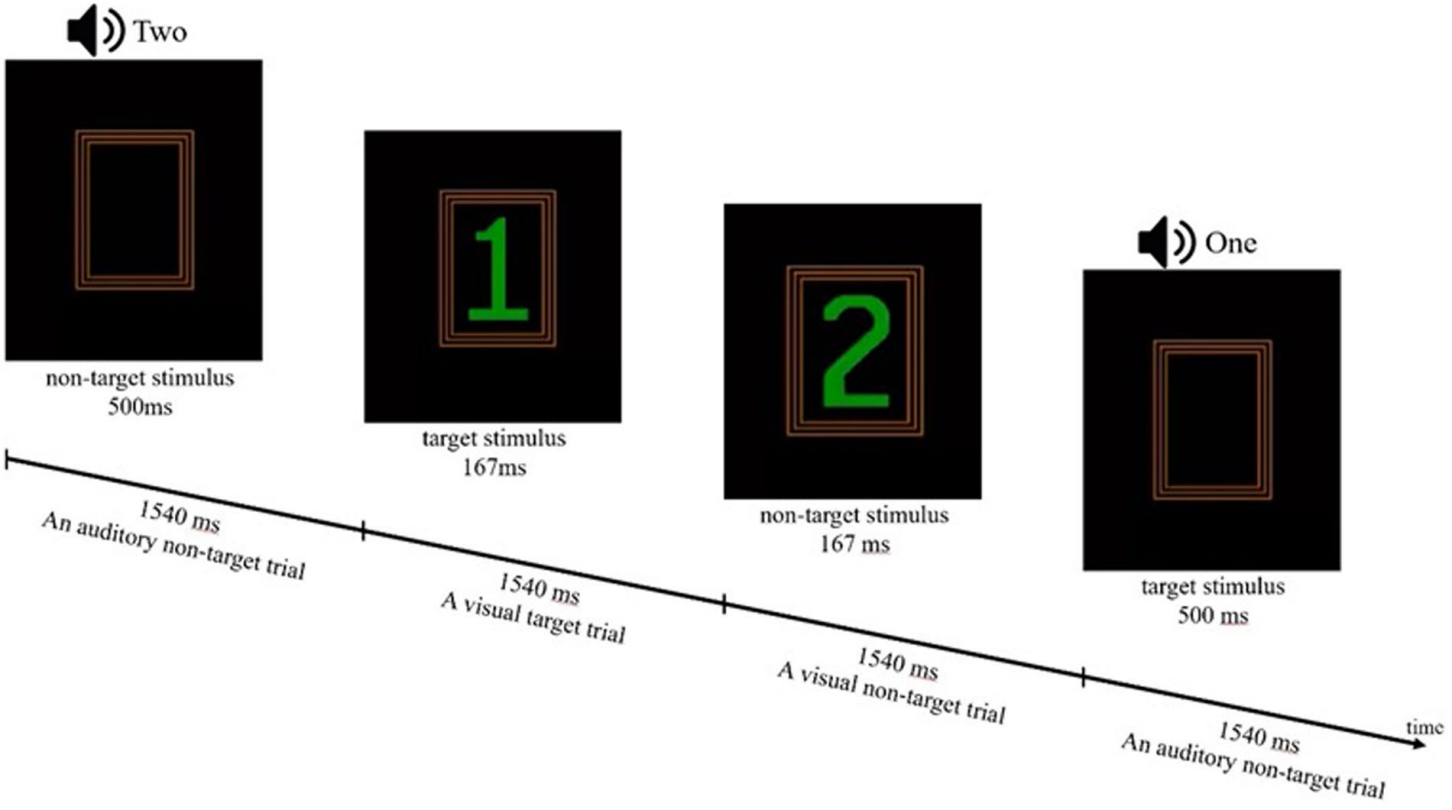
Schematic representation of the IVA-2 task. Participants responded to auditory or visual “1” targets, ignoring “2” non-targets. Each trial lasted 1540 ms. Auditory stimuli were presented for 500 ms, visual stimuli for 167 ms.

The IVA-2 involves auditory and visual stimuli: the target “1” and the non-target “2.” Participants are instructed to respond to the target “1” and to refrain from clicking for the non-target “2.” Each trial consists of one of four stimuli: a target auditory stimulus, a target visual stimulus, and their corresponding non-target counterparts. In total, there are 500 trials, each lasting 1540 ms. The duration of presentation of auditory stimuli is 500 ms and the duration of visual stimuli is 167 ms (Fig. 2). The assessment is structured into four distinct phases: warm-up, practice, main test, and cool-down and takes approximately 15 minutes.

The IVA-2 test provides scores in various categories including response control, attention, attributes, and symptoms. These scores encompass both basic scale scores and composite quotient scores. By analyzing performance nuances within auditory and visual domains, the IVA-2 serves as a comprehensive tool for evaluating attention and response control across different modalities^14^. Quotient scales provided by the IVA-2 are reported in both Table 2 and Table S2 (see Supplementary Tables).

**Table 2:** Variable Names, Scores, and Column Numbers for “**iranian_child_EEG_ERP_cognitive_dataset.csv”** file.

Variable/Feature	N° of Column	Column(s) Number(s)	Description
**ID**	1	1	Individual ID for each subject
**Education Grade**	1	2	1^st^ = First, 2^nd^ = Second, 3^rd^ = Third, 4^th^ = Fourth, 5^th^ = Fifth, 6^th^ = Sixth
**Relative**	1	3	Parent who responds to questionnaires: 1 = mother, 0 = father
**Parent Education**	1	4	0 = High school, did not graduate, 1 = High school graduate, 2 = Trade or business school, 3 = Some college, did not graduate, 4 = Junior college graduate, associate’s degree (or equivalent), 5 = College graduate, bachelor’s degree (or equivalent), 6 = Some postgraduate education, no advanced degrees, 7 = Attained 1 or more advanced degrees
**Gender**	1	5	Female, Male
**Age**	1	6	Age in months
**QEEG**	34200	7-34206	See Table S1 for details (Supplementary Tables)
Eyes Open condition	17100	7-17106	
Eyes Close condition	17100	17107-34206	
**IVA-2**	51	34207-34257	Global and Special Analyses Quotient Scale Scores. See Appendix 2 for details (Supplementary Appendices).
Language of test	1	34207	Persian
Input Device	1	34208	USB
Test Validity Checks	4	34209-34212	Valid = 1 Valid (Interpret with caution: excessive idiopathic errors) = 2 Invalid (excessive idiopathic errors) = 3
Quotient Scale Scores	45	34213-34257	See Table S2 for details (Supplementary Tables). Note that the variable is assigned a value of -9 when the information is missing or unavailable
**CSI-4**	97	34258-34354	See Table S3 for details (Supplementary Tables).
**Handedness**	10	34355-34364	1 = always right, 2 = usually right, 3 = no preference, 4 = usually left, 5 = always left
**Spatial Orientation**	2	34365-34366	0 = Not at all, … 4 = Very much
Laterality orientation	1	34365	Distinguishing left and right (no difficulty = 0, extreme difficulty = 4)
Address orientation	1	34366	Nnavigating or finding an address (no difficulty = 0, extreme difficulty = 4)
**Parent’s Math History**	23	34367-34389	Questions are in a Likert scale format in which the participant could specify the degree of struggle ratings along a continuum from 0 to 4 (no difficulty = 0, extreme difficulty = 4).
**Parent’s Reading History**	25	34390-34414	Questions are in a Likert scale format in which the participant could specify the degree of struggle ratings along a continuum from 0 to 4 (no difficulty = 0, extreme difficulty = 4).
**Working Memory Task**	39	34415-34453	See Table S4 for details (Supplementary Tables).
**Ravan Test**	3	34454-34456	See Table S5 for details (Supplementary Tables).

Please note that in Table 2, the column(s) number(s) are not Python indices; they start from 1 instead of 0. Additionally, both the start and stop of the ranges are inclusive.

### Raven Colored Progressive Matrices Test (CPM)

The Raven’s CPM is a tool commonly used to assess non-verbal intelligence, especially in children aged 5 to 11^16^. The CPM measures the ability to induce abstract relations and manage problem-solving goals in working memory^17^. The test consists of 36 items divided into three sets, using bright colors to engage children. The items are arranged by increasing difficulty^18^. The CPM might also be beneficial for individuals with reading difficulties^19,20^, physical handicaps^21^, or intellectual disabilities^22–24^.

Additionally, the CPM has been found to be stable across different cultural, ethnic, and socioeconomic groups. However, there have been observed variations in norms over time and between ethnic groups within countries^18^. The CPM has demonstrated good test-retest reliability and sensitivity to fluctuations in intellectual function^25,26^. Despite some concerns about its reliability in group settings^27^. the CPM has been found to have good factorial validity and internal consistency^28^. In this study, we utilized the computerized version of Raven’s CPM test. We provided three measures in this research: raw score, IQ scaled score, and percentile rank (refer to Table 2 and Table S5).

### Working memory

Working memory is the capability to simultaneously store and manipulate information for a limited period of time in service of goal-directed behaviors. Working memory is composed of different components: the central executive, which controls and coordinates the other components; the phonological loop, which processes and stores verbal information, such as words and sounds; and the visuospatial sketchpad, which handles visual and spatial information, such as images and mental maps^29^.

Although phonological processing deficits are accepted as the most dominant concept of the cognitive origin of dyslexia, working memory impairments are also mentioned as one of the most affected cognitive domains. Moreover, dyscalculia is an impairment in processing numerical magnitudes, although deficits in memory, attention, and processing speed have also been reported^12^. Working memory is regarded as a significant predictor of academic achievement in areas like reading and mathematics and has been suggested to be the cognitive domain that is consistently most affected in SLD children^30^.

To investigate working memory in more detail, we developed a modified version of the task inspired by recent studies^31–33^. This task specifically targets working memory by integrating visual-spatial and phonological processing through the use of both numerical and non-numerical stimuli. The task consists of two distinct blocks, each designed to assess working memory with different types of stimuli.

In the first block, participants completed 70 trials where they were presented with one-syllable, non-numerical Persian words (e.g., bag, moon). The use of one-syllable words controlled the phonological load on working memory, ensuring that participants focused primarily on the task demands. This approach also standardized conditions, ensured age-appropriateness, and maintained consistency across the experiment.

**Fig. 3:**
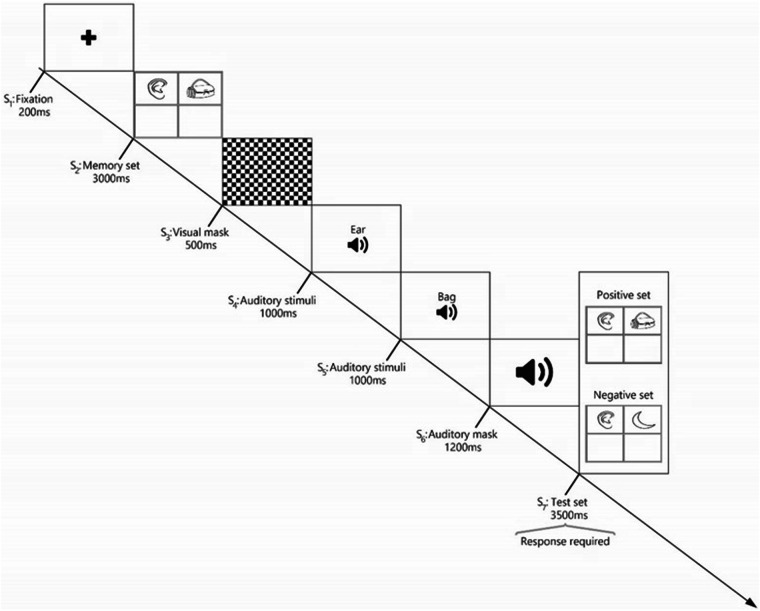
A visual illustration of a trial in the non-numerical block of the working memory task. Initially, participants are presented with a memory set containing two images within a four-grid square was presented (S_2_), followed by a visual mask (S_3_). Participants are then required to memorize the subsequent auditory stimuli (S_4_ and S_5_), followed by an auditory mask (S_6_). In the final step (S_7_:test set), participants are asked to determine whether the presented pictures matched the memory set (positive set) or not (negative set).

As illustrated in Fig. 3, each trial in the first block consisted of seven stimuli. Initially, a fixation point (S_1_) was presented for 200 milliseconds (ms) followed by the presentation of two pictures of one-syllable words within a four-grid square for 3000 ms (S_2_). Participants were required to memorize these pictures and their locations (which served as the memory set). A visual mask (S_3_) then appeared for 500 ms to eliminate sensory memory traces. Sequentially presented auditory word stimuli (S_4_ and S_5_), recorded under controlled conditions, followed. Participants were also required to memorize these words. After a blank white display with white noise (S_6_), a test set step (S_7_) presented two pictures and Participants were required to determine whether these pictures matched the previously memorized pictures from the memory set. Each block lasted approximately 15 minutes.

The experiment consisted of 70 trials including 30 positive sets and 40 negative sets. The terms “positive” and “negative” refer to the compatibility of pictures in the test set (S_7_) with those in the memory set (S_2_). In the positive sets, the pictures were identical to those that memorized earlier. Conversely, in the negative set pictures differed from the memory set in one of four possible ways: 1) One picture location was changed, 2) Two picture locations were changed, 3) one content alteration was made, or 4) two content alterations were made. Participants were asked to respond quickly and accurately, indicating whether the pictures matched their memory. All trials, both positive and negative, were presented in a pseudo-randomized order, ensuring a consistent format for all participants. This structure facilitated a comprehensive assessment of the participants’ working memory capabilities.

**Fig. 4:**
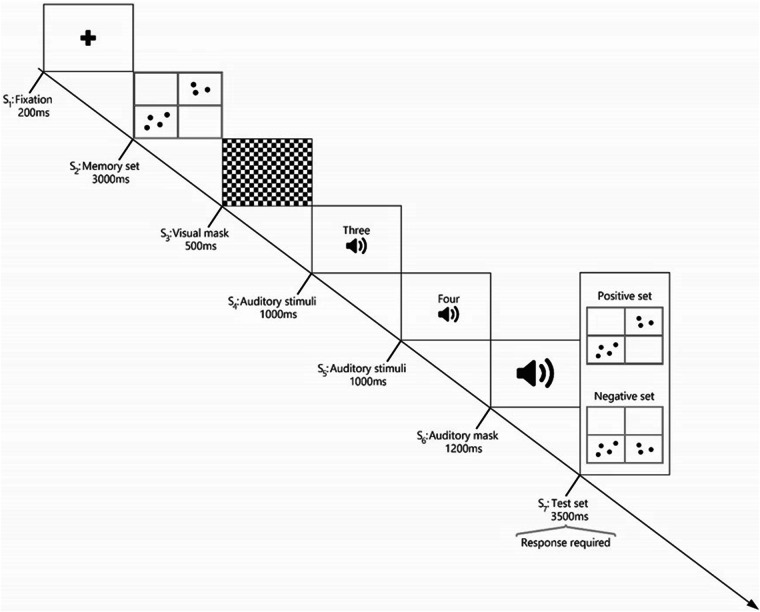
A visual depiction of the second block (numerical block) in the working memory task. Initially, participants viewed a memory set consisting of two arrangements of dots representing numbers within a four-grid square (S_2_), followed by a visual mask. Participants were then tasked with memorizing the subsequent auditory stimuli. In the final stage (S_7_: test set), participants determined whether the displayed numbers corresponded to the memory set (positive set) or not (negative set).

The structure of the second block mirrored that of the first block in terms of task structure, the number of trials, conditions, and instructions. However, the focus shifted to numbers (1 to 4) and dice-distributed dots (see Fig. 4). Participants were once again asked to determine if the test set (S_2_) corresponded to the memory set (S_7_). To minimize potential learning effects resulting from a fixed presentation order, the blocks were presented in a random sequence (see Fig. 4).

The experiment utilized a 15-inch monitor to present stimuli to participants seated approximately 40 cm away in a dimly lit room. Participants were instructed to respond as quickly and accurately as possible. Prior to the main experiment, practice trials ensured a thorough understanding of the task. No performance feedback was provided during the main experiment. The task was administered using PsyTask software (v. 1.57.21 Mitsar Ltd.). This novel working memory task not only advances our understanding of working memory processes but also provides a versatile tool for future research in this domain.

The behavioral dependent variables of interest in the working memory task were hit rate, false alarm rate and d-prime (d’). These measures are presented in their raw state, as reported in Table 2 and Table S4.

#### Hit rate

**Fig. 5:**
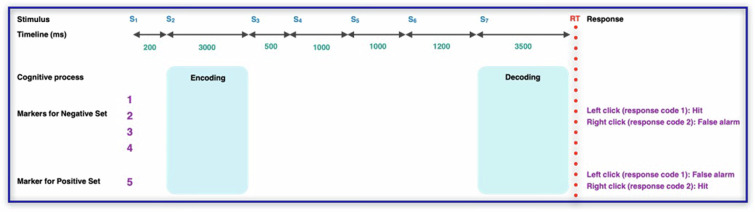
The experimental task and its corresponding markers for ERP analysis. The experiment comprised 70 trials, divided into 40 negative trials and 30 positive trials. In negative set trials, the test picture (S_7_) differed from the memorized picture (S_2_) in one of four ways: marker 1 (one location change), marker 2 (two location changes), marker 3 (one content alteration), or marker 4 (two content alterations). For negative set trials, marker 1 in the response channel indicates a hit response, while marker 2 signifies a false alarm. Conversely, in positive trials, the test picture was identical to the memorized picture (marker 5). Here, marker 1 in the response channel denotes a false alarm, and marker 2 indicates a hit response.

This refers to the proportion of correct responses where the participant correctly identified a target item they were supposed to remember (see Fig. 5).

- Hit rate is calculated for each negative set condition, positive set condition and for each block separately.
- The formula is: Hit rate = (number of correct trials in condition X) / (total number of trials in condition X).
- To obtain an overall hit rate for the negative set conditions within each block, the hit rates are averaged: Hit rate (negative set) = (Hit rate condition 1 + Hit rate condition 2 + Hit rate condition 3 + Hit rate condition 4)/4.

#### False alarm rate

This is the proportion of incorrect responses where the participant mistakenly identified a non-target item as a target item (see Fig. 5).

- The false alarm rate is calculated in the same way as the hit rate, but for incorrect responses.
- False alarm rate = (false trials in condition X) / (total trials in condition X).
- Similar to the hit rate, the false alarm rate for the negative set conditions within each block is calculated by averaging the false alarm rates from each condition.

#### D-prime (d’)

Signal Detection Theory (SDT) offers a sophisticated approach to assessing performance in cognitive tasks (e. g. working memory task). Unlike traditional measures like hit rate and false alarm rate, which provide limited insights into a participant’s ability to discriminate between targets (signal) and distractors (noise), d-prime offers a more comprehensive evaluation^34^. While the hit rate quantifies the proportion of correctly identified targets and the false alarm rate reflects the proportion of incorrectly identified distractors, neither measure alone provides a complete picture of a participant’s discrimination ability.

D-prime integrates both the hit rate and false alarm rate into a single metric, offering a more nuanced understanding of a participant’s performance. A higher d-prime value indicates a stronger ability to distinguish between targets and distractors^35^. This implies better sensitivity and accuracy in working memory task.

- D-prime is calculated for each condition (positive set and negative set) within a block using the following formula: d’ = z (Hit Rate) - z (False Alarm Rate)^36^. For example, if the hit rate is 0.8 and the false alarm rate is 0.2, the corresponding z-scores are approximately 0.84 and -0.84, respectively. Thus, d’ = 0.84 - (-0.84) = 1.68, indicating a relatively strong ability to discriminate between targets and distractors.
- To obtain an average d’ for the negative set condition within a block, individual d’ values are averaged: $d{\prime} \left({negative\; set}\right)=\,\mathop{\sum }\limits_{i=1}^{4}d{\prime} ({{condition}}_{i})/4$, where ∑ represents the sum.
- Similarly, to obtain a total d’ for each block is obtained by averaging the d’ values from the negative set and positive set conditions: d’ (block) = (d’ negative set + d’ positive set)/2.
- Finally, grand total d’ for the entire memory task is calculated by averaging the d’ values from each block: d’ (total) = (d’ block 1 + d’ block 2)/2.

## EEG and ERP Data Collection

### Electrode placement

The EEG cap (Electro-Cap International Inc., OH, USA) was then carefully placed on the child’s head according to the international 10-20 system. EEG data were collected from 19 scalp electrodes (Fp1, Fp2, F3, F4, C3, C4, P3, P4, O1, O2, F7, F8, T3, T4, T5, T6, Fz, Cz, Pz). Ground and reference electrodes (linked ears) were meticulously cleaned. Linked ears served as the reference (left and right earlobes) and the ground electrode was placed at the AFz. A Mitsar 201 amplifier and WinEEG software were employed for data acquisition, with a sampling rate of 250 Hz and a bandpass filter of 0.16 Hz - 70 Hz. To eliminate line noise, a notch filter at 45-55 Hz and 95-105 Hz was noise applied. The conductive gel was then applied to the electrodes, ensuring that electrode impedance was remained below 10 kΩ for each electrode. The reference electrodes were also carefully attached.

Participants were seated in a quiet, well-lit, and sound-attenuated experimental environment, where chairs and tables were adjusted to suit the children’s comfort. To minimize anxiety, parents were permitted to be present in the recording room, positioned discreetly in a corner. Researchers patiently answered any questions from both the children and their parents. The EEG recording consisted of two stages. First, a resting-state stage involved two 4-minute recordings: one with eyes closed and one with eyes open. To minimize eye movement, participants were instructed to fixate on a central point during the open-eye condition and to keep their eyes closed and imagine a fixed point during the closed-eye condition. Second, ERP recording in which participants completed our modified working memory task while EEG data was simultaneously recorded.

Participants received instructions for the memory task and were asked to minimize movement, maintaining stillness during the task presentation. The memory task was visually presented through the PsyTask (Mitsar Ltd.) slave system, while a research associate monitored and recorded EEG data using the master system. The technician continuously monitored the subject’s recordings to prevent contamination of the EEG signal by electromyogram interference, fluctuations in direct current caused by sweating, and potential drowsiness.

### QEEG extraction

Quantitative Electroencephalography (QEEG) represents a contemporary approach to EEG analysis, involving the extraction of features from EEG signals to provide a detailed understanding of brain dynamics. These extracted features allow for the analysis of specific frequency bands, signal complexity, functional connectivity, and network-based brain activity^37^. QEEG utilizes mathematical algorithms to process and analyze digital EEG recordings, transforming raw brainwave activity into quantitative metrics that can be used for clinical or research purposes.

For our study, we employed NeuroGuide software (version 3.2.1, Applied Neuroscience, Inc.) to extract QEEG values. The preprocessing phase began with an extensive visual inspection of the EEG data, conducted to ensure the removal of unwanted artifacts. This included a two-tiered editing approach: automatic detection algorithms were initially applied to flag obvious biological artifacts (such as eye blinks, muscle movements, and heartbeats) and environmental noise (e.g. electrical interference). Subsequently, a manual review was performed to ensure that these artifacts were accurately identified and excluded, minimizing any impact on the data quality and ensuring the integrity of the analysis.

Once artifact-free EEG segments were selected, they were transformed from the time domain into the frequency domain using the Fast Fourier Transform (FFT) algorithm. This transformation allowed for a comprehensive spectral analysis, enabling the decomposition of EEG signals into distinct frequency components. The extracted QEEG values were then categorized across several frequency bands, including Delta (1.0–4.0 Hz), Theta (4.0–8.0 Hz), Alpha (8.0–12.0 Hz), Beta (12.0–25.0 Hz), High Beta (25.0–30.0 Hz), Gamma (30.0–40.0 Hz), High Gamma (40.0–50.0 Hz), and sub-bands such as Alpha 1 (8.0–10.0 Hz), Alpha 2 (10.0–12.0 Hz), Beta 1 (12.0–15.0 Hz), Beta 2 (15.0–18.0 Hz), Beta 3 (18.0–25.0 Hz), Gamma 1 (30.0–35.0 Hz), and Gamma 2 (35.0–40.0 Hz).

Seven key features were extracted from the resting-state EEG data under both eyes open and eyes closed conditions, each providing unique insights into the brain’s functional state. These features include: absolute power (the total power within each frequency band, reflecting the overall strength of neural oscillations), relative power (the proportion of power in a given frequency band relative to the total power across all bands, highlighting the distribution of brain activity), power ratio (ratios between different frequency bands, which can reveal imbalances in brainwave activity), peak frequency (the dominant frequency within each band, indicating the rate at which neuronal assemblies are firing), amplitude asymmetry (differences in signal amplitude between homologous brain regions, often used to assess hemispheric imbalances), coherence (a measure of functional connectivity, representing the degree of synchronization between signals from different brain regions), and phase lag (the temporal delay between oscillations from separate brain areas, providing insights into the directionality and efficiency of neural communication).

To aid in the interpretation and clinical relevance of these features, z-scores were calculated using the NeuroGuide software’s normative database. These z-scores allowed for a standardized comparison of an individual’s QEEG values against age-matched norms, helping to identify deviations from typical brain activity patterns. The use of these normative comparisons facilitated a more objective and comprehensive understanding of the brain’s functional state Raw data for all indicators are reported alongside their age-specific z-scores (see Table 2 and Table S1 in Supplementary Tables).

## Questionnaires

### Child Symptom Inventory–4 (CSI–4)

The CSI-4 is a widely used behavioral rating scale designed to assess symptoms associated with childhood disorders as defined by the DSM-IV criteria. Research supports the reliability and validity of CSI–4 across diverse populations and settings, making it a valuable tool for assessing childhood behavioral and emotional disorders^38^. In this study, we employed the Persian version of the parent questionnaire, comprising 97 items. All of these scores are reported in raw form (see Table 2 and Table S3 for data information and Appendix 1 (Supplementary Appendices) for CSI–4 items).

### Edinburgh Handedness Inventory (EHI)

We used the EHI to evaluate children’s handedness. This scale, consisting 10 items, measures an individual’s preference for using their right or left hand in everyday activities^39^^,40^. Additionally, we included two supplementary items to assess the children’s ability to differentiate between right and left and their navigational proficiency. All of these scores are reported in raw form (see Table 2 for data information and Appendix 2 (Supplementary Appendices) for EHI items).

### Adult Reading History Questionnaire (ARHQ)

The influence of familial reading history on a child’s literacy development and reading achievements has been extensively studied. Research indicates that children from families with a history of reading difficulties may exhibit poorer letter-word knowledge and phonological awareness^41,42^. These foundational skills, crucial for future reading success, are typically established before formal education begins^43^. Additionally, children with a family history of reading difficulties often struggle with word recognition by the time they enter elementary school, impacting their ability to benefit from explicit reading instruction^44,45^. The ARHQ^46^ is a reliable screening tool used to assess the risk of dyslexia in adults. The questionnaire consists of 23 items measured on a five-point Likert scale, with higher scores on the ARHQ correspond to a greater likelihood of a familial reading difficulties. By providing insights into the home literacy environment, the ARHQ aids in tailoring interventions to support children’s reading outcomes based on a family history assessment. All of these scores are reported in raw form (see Table 2 for data information and Appendix 3 (Supplementary Appendices) for ARHQ items).

### Adult Arithmetic History Questionnaire (AAHQ)

Emerging research suggests a link between family history of math difficulties and a child’s performance in math. Similar to how strong word recognition skills are fundamental for complex reading tasks, arithmetic calculation proficiency is a core skill for math success. This proficiency encompasses the ability to solve single-digit and often multi-digit addition and subtraction problems^47,48^.

The AAHQ^49^ is a self-report questionnaire modeled after the ARHQ.It assess the history of arithmetical difficulties in adults^46^ exploring areas such as basic number knowledge, challenges in learning foundational arithmetic and retrieving arithmetic facts, and the everyday use of numbers and arithmetic. Utilizing a 25-item, five-point Likert scale, higher AAHQ scores indicate a greater likelihood of having math difficulties. All of these scores are reported in raw form (see Table 2 for data information and Appendix 4 (Supplementary Appendices) for AAHQ items).

## Data Records

To support and facilitate ongoing research endeavors, we have made dataset available under controlled access at *Synapse.org* repository^50^. Researchers interested in accessing the dataset can submit a request following the outlined procedures to ensure compliance with ethical and privacy considerations (see *Usage Notes* section). This comprehensive dataset includes raw EEG/ERP files, all extracted features from QEEG and ERP, cognitive tasks results, and questionnaires responses. Additionally, Python codes were developed to organize and process the extracted features for researchers’ use. The dataset is structured in three folders as follows:

1. Features extracted from all data:
  A detailed *Tables.docx* file provides descriptions of the sample demographic, variable names, QEEG features, test scores, column numbers, and associated details.
  This dataset is presented as a large matrix file in CSV format, comprising 100 rows (participants) and 34458 columns (variables). The Columns Include:
    **Participant ID:** A unique identifier for each participant (1 column).
    **Demographic data:** Age, gender, and other related information (5 columns).
    **QEEG Features:** Raw and age-adjusted standard scores from rest-state conditions:
      Eyes Open (EO): 17,100 columns.
      Eyes Closed (EC): 17,100 columns.
    **Cognitive Measures:**
      IVA-2 age-adjusted quotient scores (51 columns).
      CSI-4 questionnaire raw scores across clinical scales (97 columns).
      Handedness inventory raw scores (10 columns).
      Spatial orientation raw scores (2 columns).
      Parent’s math history raw scores (23 columns).
      Parent’s reading history raw scores (25 columns).
      Ravan ‘s Progressive Matrices: raw score, age-adjusted IQ, and percentile rank (3 columns).
2. **Raw EEG/ERP data:**
  Raw EEG/ERP recordings in EDF format are provided for all 100 participants within compressed.RAR folders. These include the following:

- Rest-state EEG during the Eyes Closed (EC) condition.
- Rest-state EEG during the Eyes Open (EO) condition.
- ERP recordings during the numerical block of the working memory task.
- ERP recordings during the non-numerical block of the working memory task.

**Note:** All EEG records have been anonymized by removing personal information to prevent identification of the participants. Participant IDs are consistent across the CSV file and the raw EEG/ERP data files.

3. **Software Package:**

A Python-based toolkit is included to streamline data analysis, offering functions for reading and filtering cognitive data from the CSV file, along with tools for exporting processed results to facilitate further analysis. This toolkit is designed to assist researchers in efficiently handling the cognitive and EEG/ERP datasets.

## Technical Validation

### Data quality control and validation for behavioral data

The quality of the behavioral data was ensured through rigorous task design, clear participant instructions, and careful data cleaning. Outliers and errors were identified and removed to maintain data integrity. All data were collected by trained experts under the supervision of a board-certified clinical psychologist. After input, data were organized by a research and development team at the Imâge Brain Institute using statistical summary tools to ensure quality control.

All tests and questionnaires used in this study, except for our novel working memory task, are validated and standardized. Previous studies^51^ have shown that working memory can mediate the relationship between fluid intelligence, as measured by Raven’s Matrices. As expected, a significant positive correlation was found between performance on our working memory task (i.e. d-prime) and raw score of Raven (r = 0.406, p < 0.0001), supporting the validity of our task. Furthermore, given the expected developmental increase in working memory capacity during ages 6-11, and considering that age was recorded in months to capture finer-grained developmental differences, the significant strong positive correlation between performance on the working memory task and age (in month) (r = 0.629, p < 0.0001) further validates our task’s sensitivity to individual differences in working memory.

### Data quality control and validation for EEG

**Fig. 6:**
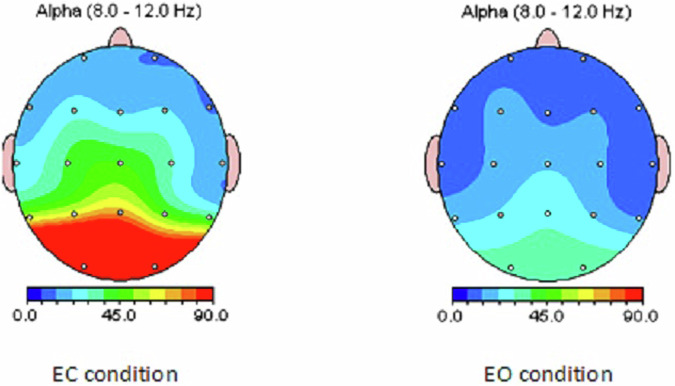
Alpha Power Distribution: Comparison between Eyes-Closed (EC) and Eyes-Open (EO) Conditions. Alpha power is suppressed in EO condition, particularly at posterior sites.

To ensure the highest quality of our EEG data, we employed a comprehensive multi-step approach:

1. Data Acquisition:
  The EEG cap was carefully placed on the child’s head, ensuring proper fit. Additionally, the distance between the nasion and inion landmarks was measured to ensure accurate placement of the EEG cap according to the international 10-20 system.
  We carefully monitored electrode impedance levels to minimize noise and artifacts. Impedance values were maintained below 10 kΩ to ensure optimal signal quality.
  The experimental environment was carefully controlled to reduce the impact of external factors, such as noise, temperature, and lighting, on the EEG recordings.
  The technicians documented ocular movements and other relevant events during the recording sessions.
  All technicians were trained experts, qualified in EEG recording procedures.
2. **Statistical Quality Control:** Alpha power suppression is a well-established EEG phenomenon, particularly noticeable at posterior sites during the transition from an eyes-closed to an eyes-open condition^52^. To visualize these changes, we created a topographic map of alpha power (Fig. 6). This map clearly illustrates a considerable increase in alpha power in the EC condition compared to the EO condition, especially in posterior brain regions.

### Data quality control and validation for ERP

Behavioral assessments offer valuable insights into accuracy and reaction time but may not fully elucidate underlying neurocognitive processes. EEG combined with ERPs, with their high temporal resolution (millisecond), offer a valuable tool for investigating these processes capturing neural activity associated with various cognitive functions, such as sensory encoding, inhibition, and working memory^53^. Previous studies employing ERP analysis in working memory tasks have demonstrated that children with learning disabilities exhibit altered component characteristics, suggesting an increased cognitive effort^31,54^.

### ERP extraction and preprocessing

ERP pre-processing was conducted using the EEGLAB toolbox (ver. R2023a) in MATLAB (The MathWorks, Inc., United States, ver. R2023a), following established protocols and Makoto’s pipelines (Miyakoshi, 2018). EDF + files containing 20 channels (19 EEG, 1 event) were imported. A band-pass filter (0.5-30 Hz) and notch filters (45-55 Hz, 95-105 Hz) were applied to remove noise. Artifact Subspace Reconstruction (ASR) was used to eliminate large artifacts, and Independent Component Analysis (ICA) was employed to remove non-brain sources. Components with a brain source probability exceeding 70% were retained.

### ERP Computation

ERPs were computed for each block (numerical and non-numerical). Data were locked to stimulus 1 and averaged across all trials. Data were further averaged according to task condition (negative or positive set). ERPs were computed based on markers 1-4 for negative sets and marker 5 for positive sets (see Fig. 5). ERP waveforms were extracted for all 19 electrodes, covering the entire trial duration.

### ERP Analysis

**Fig. 7:**
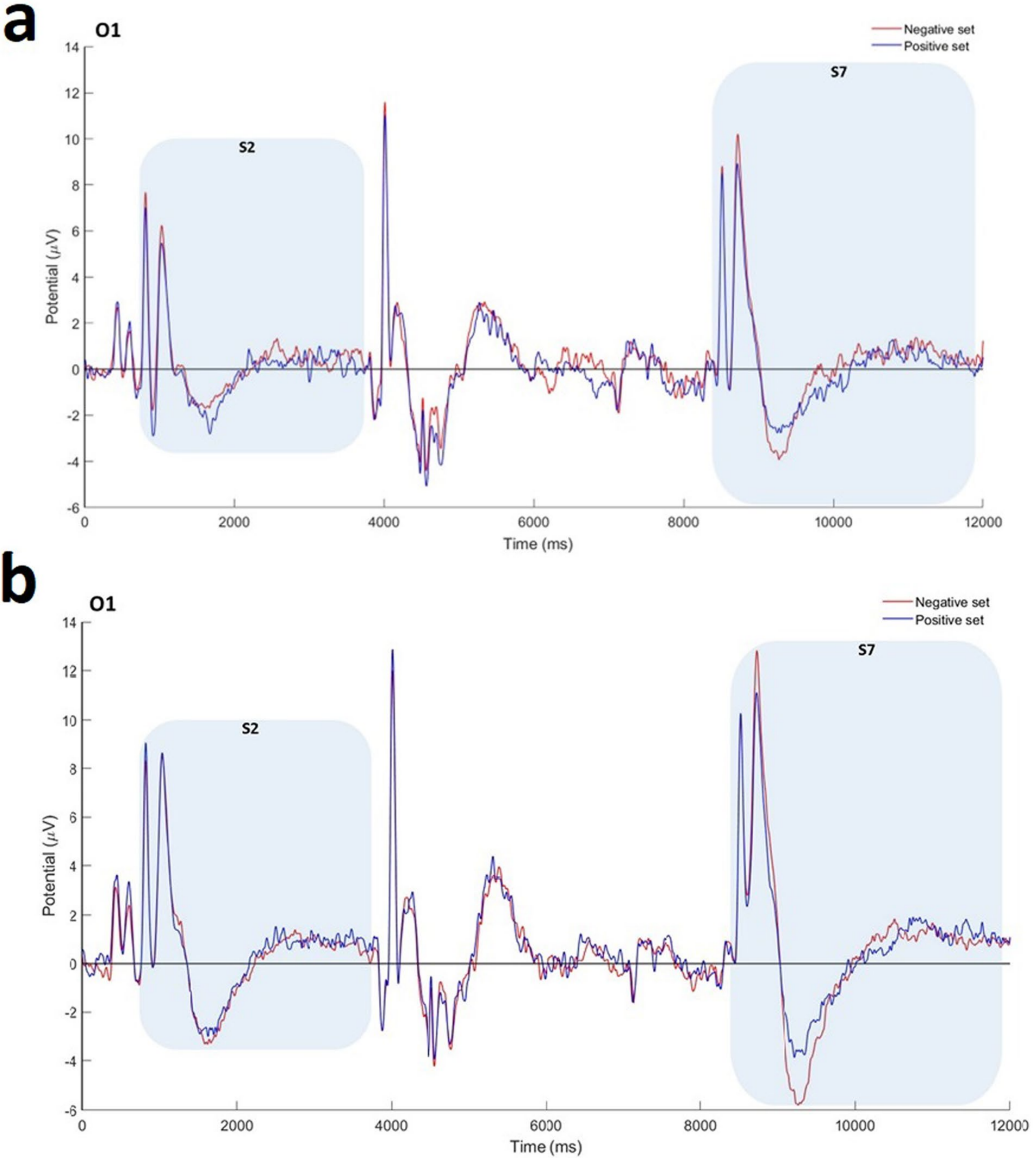
A visual depiction of grand average ERP components elicited during a working memory task. Lines represent average brain activity across participants for the entire trial duration for the numerical block (**a**) and the non-numerical block (**b**). Colors distinguish between experimental conditions: positive sets (blue) and negative sets (red). S_2_ and S_7_ markers indicate the memory set (encoding) and test set phases of the working memory task, respectively. The amplitude of the LPN component was significantly greater for negative sets compared to positive sets [F(1,99) = 6.132, p = 0.026].

Fig. 7 illustrates ERP activity at the O1 electrode for numerical and non-numerical blocks and conditions (positive and negative sets). ERP patterns for each block revealed activation of working memory components during both memory encoding and test steps for 100 participants. Major components (e.g., P300 and late Posterior Negativity (LPN)) were identified. Notably, the amplitude of LPN was significantly greater for negative sets compared to positive sets [F(1, 99) = 6.132, p = 0.026]. These initial findings suggest potential neural signatures related to type of stimulus (numerical vs. non-numerical) and set type (positive vs. negative) within a working memory task. Aligned with LPN literature^55^, a significant difference in LPN amplitude between conditions suggests that our task successfully elicited differential neural responses. This indicates that our experimental paradigm was sensitive enough to capture meaningful cognitive processes.

Further investigation and more robust statistical comparisons are warranted to corroborate these initial observations.

## Usage Notes

Related information and comprehensive instructions for data usage are provided in the “***Accessing the Dataset****”* **wiki** tab of the project^50^. Researchers interested in accessing the dataset should complete the following steps:

1. Register for a Synapse account (www.synapse.org).
2. Become a Synapse *Certified* and *Validated* User.
3. Request to Join “*Iranian 6-11 Years Population-Based EEG, ERP, and Cognition Dataset Users*” Team.To join, the researcher must provide their name and institution in the text box, include their agreement to the Conditions of Use and Intended Data Use Statement, and click the Send Request button. An administrator will review requests within 48 working hours, and confirmation will be sent via email upon approval.

## Supplementary information

Supplementary Tables

Supplementary Appendices

## Data Availability

The custom code (*cognitive_data_utils.py*) and data-processing workflows (*Read Me.txt*) are available in the ***Software Package*** folder, located within the **Files** tab of the project. These resources can be accessed after completing the required steps (see Usage Notes section) via the following link: https://www.synapse.org/Synapse:syn64145789. Follow these steps to utilize the provided toolkit in software package effectively: 1. Clone this repository to your local machine. 2. Ensure you have Python installed on your system. 3. Install the required dependencies by running pip install pandas. It is important to note that in Python, indices start with 0. Therefore, index = 0 in axis = 1 corresponds to column number 1. ***Downstream Processing Steps:*** Suggested steps for downstream processing and relevant procedures, are outlined as follow: 1. Import the required functions from the toolkit 2. Read the cognitive data from the CSV file 3. Apply filters to the data based on specified criteria 4. Export the filtered data to a new CSV file
