# Supplementary material for: Iranian 6-11 years age population-based EEG, ERP, and cognition dataset
Source: Sci Data. 2025 Feb 22;12:319. doi: 10.1038/s41597-025-04624-6 (PMC11846862; doi:10.1038/s41597-025-04624-6)
Supplement: Supplementary file 1 — Supplementary Tables [file 41597_2025_4624_MOESM1_ESM.pdf]

Tables

Table S1. Eye Open and Eye Closed QEEG Features

Table S2. Global and Special Analyses Quotient Scales provided by IVA-2

Table S3. Child Symptom Inventory–4 Scales

Table S4. Working Memory Task Scales

Table S5. Ravan Test Scales

Table S1. Eye Open and Eye Closed QEEG Features

Column N° in Data file	Variable Name	Scale	Scale Description
**Rest state EO condition**			
7-272	('abs_eo_FP1-LE', 'Delta'), … ('abs_eo_Pz-LE', ' Gamma2')	FFT Absolute Power	19 electrode × 14 Frequency Band, with this order: Fp1Delta, Fp1Theta, Fp1Alpha, … PzBeta3, PzGamma1, PzGamma2
273-462	('z_abs_eo_FP1-LE', 'Delta'), … ('z_abs_eo_Pz-LE', 'Beta3')	Z score Absolute Power	19 electrode × 10 Frequency Band, with this order: Fp1Delta, Fp1Theta, Fp1Alpha, … PzBeta3
463-728	('rel_eo_FP1-LE', 'Delta'), … ('rel_eo_Pz-LE', 'Gamma2')	FFT Relative Power	19 electrode × 14 Frequency Band, with this order: Fp1Delta, Fp1Theta, Fp1Alpha, … PzBeta3, PzGamma1, PzGamma2
729-918	('z_rel_eo_FP1-LE', 'Delta'), … ('z_rel_eo_Pz-LE', 'Beta3')	Z score Relative Power	19 electrode × 10 Frequency Band, with this order: Fp1Delta, Fp1Theta, Fp1Alpha, … PzBeta3
919-1108	('rat_eo_FP1-LE', 'Delta / Theta'), … ('rat_eo_Pz-LE', ' Beta / High Beta ')	FFT Power Ratio	19 electrode × 10 ratio Frequency Band, with this order: Fp1Delta / Theta, Fp1Delta / Alpha, Fp1Delta / Beta, Fp1 Delta / High Beta, Fp1 Theta / Alpha, … Pz Beta / High Beta
1109-1298	('z_rat_eo_FP1-LE', 'Delta / Theta'), … ('z_rat_eo_Pz-LE', ' Beta / High Beta)	Z score Power Ratio	19 electrode × 10 ratio Frequency Band, with this order: Fp1Delta / Theta, Fp1Delta / Alpha, Fp1Delta / Beta, Fp1 Delta / High Beta, Fp1 Theta / Alpha, … Pz Beta / High Beta
1299-2248	('abs_1_eo_FP1-LE', '1 Hz'), … ('abs_1_eo_Pz-LE', '50 Hz')	FFT Absolute Power 1 Hz	19 electrodes × [1-50] Hz, with this order: Fp1-1Hz, Fp1-2(Hz), Fp1-3(Hz), … Pz-49(Hz), Pz-50(Hz).
2249-2818	('z_abs_1_eo_FP1-LE', '1 Hz'), … ('z_abs_1_eo_Pz-LE', '30 Hz')	Z score Absolute Power 1 (Hz)	19 electrodes × [1-30] (Hz), with this order: Fp1-1(Hz), Fp1-2(Hz), Fp1-3(Hz), … Pz-29(Hz), Pz-30(Hz).
2819-3768	('rel_1_eo_FP1-LE', '1 Hz'), … ('rel_1_eo_Pz-LE', '50 Hz')	FFT Ralative Power 1 (Hz)	19 electrodes × [1-50] (Hz), with this order: Fp1-1(Hz), Fp1-2(Hz), Fp1-3(Hz), … Pz-49(Hz), Pz-50(Hz).
3769-4338	('z_rel_1_eo_FP1-LE', '1 Hz'), … ('z_rel_1_eo_Pz-LE', '30 Hz')	Z score Relative Power 1 (Hz)	19 electrodes × [1-30] (Hz), with this order: Fp1-1(Hz), Fp1-2(Hz), Fp1-3(Hz), … Pz-29(Hz), Pz-30(Hz).
4339-4604	('peak_eo_FP1-LE', 'Delta'), … ('peak_eo_Pz-LE', 'Gamma 2')	FFT Peak frequency	19 electrode × 14 Frequency Band, with this order: Fp1Delta, Fp1Theta, Fp1Alpha, … PzBeta3, PzGamma1, PzGamma2
4605-4794	('z_peak_eo_FP1-LE', 'Delta'), … ('z_peak_eo_Pz-LE', 'Beta 3')	Z score Peak frequency	19 electrode × 10 Frequency Band, with this order: Fp1Delta, Fp1Theta, Fp1Alpha, … PzBeta3
4795-7188	('amp_eo_FP1 FP2', 'Delta'), … ('amp_eo_Cz Pz', 'Gamma 2')	FFT Amplitude Asymmetry	((19 Electrodes × 18 Electrodes)/2) × 14 Frequency Band, with this order: FP1 FP2Delta, FP1 FP2Theta, FP1 FP2Alpha, … Cz PzGamma1, Cz PzGamma2
7189-8898	('z_amp_eo_FP1 FP2', 'Delta'), … ('z_amp_eo_Cz Pz', 'Beta 3')	Z score Amplitude Asymmetry	((19 Electrodes × 18 Electrodes)/2) × 10 Frequency Band, with this order: FP1 FP2Delta, FP1 FP2Theta, FP1 FP2Alpha, … Cz PzBeta2, Cz PzBeta3
8899-11292	('coh_eo_FP1 FP2', 'Delta'), … ('coh_eo_Cz Pz', 'Gamma 2')	FFT Coherence	((19 Electrodes × 18 Electrodes)/2) × 14 Frequency Band, with this order: FP1 FP2Delta, FP1 FP2Theta, FP1 FP2Alpha, … Cz PzGamma1, Cz PzGamma2
11293-1302	('z_coh_eo_FP1 FP2', 'Delta'), … ('z_coh_eo_Cz Pz', 'Beta 3')	Z score Coherence	((19 Electrodes × 18 Electrodes)/2) × 10 Frequency Band, with this order: FP1 FP2Delta, FP1 FP2Theta, FP1 FP2Alpha, … Cz PzBeta2, Cz PzBeta3
13003-15396	('phl_eo_FP1 FP2', 'Delta'), … ('phl_eo_Cz Pz', 'Gamma 2')	FFT Phase lag	((19 Electrodes × 18 Electrodes)/2) × 14 Frequency Band, with this order: FP1 FP2Delta, FP1 FP2Theta, FP1 FP2Alpha, … Cz PzGamma1, Cz PzGamma2
15397-17106	('z_phl_eo_FP1 FP2', 'Delta'), … ('z_phl_eo_Pz FP2', 'Beta3')	Z score Phase lag	((19 Electrodes × 18 Electrodes)/2) × 10 Frequency Band, with this order: FP1 FP2Delta, FP1 FP2Theta, FP1 FP2Alpha, … Cz PzBeta2, Cz PzBeta3
**Rest state EC condition**			
17107-17372	('abs_ec_FP1-LE', 'Delta'), … ('abs_ec_Pz-LE', ' Gamma2')	FFT Absolute Power	19 electrode × 14 Frequency Band, with this order: Fp1Delta, Fp1Theta, Fp1Alpha, … PzBeta3, PzGamma1, PzGamma2
17373-17562	('z_abs_ec_FP1-LE', 'Delta'), … ('z_abs_ec_Pz-LE', 'Beta3')	Z score Absolute Power	19 electrode × 10 Frequency Band, with this order: Fp1Delta, Fp1Theta, Fp1Alpha, … PzBeta3
17564-17828	('rel_ec_FP1-LE', 'Delta'), … ('rel_ec_Pz-LE', 'Gamma2')	FFT Relative Power	19 electrode × 14 Frequency Band, with this order: Fp1Delta, Fp1Theta, Fp1Alpha, … PzBeta3, PzGamma1, PzGamma2
17829-18018	('z_rel_ec_FP1-LE', 'Delta'), … ('z_rel_ec_Pz-LE', 'Beta3')	Z score Relative Power	19 electrode × 10 Frequency Band, with this order: Fp1Delta, Fp1Theta, Fp1Alpha, … PzBeta3
18019-18208	('rat_ec_FP1-LE', 'Delta / Theta'), … ('rat_ec_Pz-LE', ' Beta / High Beta ')	FFT Power Ratio	19 electrode × 10 ratio Frequency Band, with this order: Fp1Delta / Theta, Fp1Delta / Alpha, Fp1Delta / Beta, Fp1 Delta / High Beta, Fp1 Theta / Alpha, … Pz Beta / High Beta
18209-18398	('z_rat_ec_FP1-LE', 'Delta / Theta'), … ('z_rat_ec_Pz-LE', ' Beta / High Beta)	Z score Power Ratio	19 electrode × 10 ratio Frequency Band, with this order:

			Fp1Delta / Theta, Fp1Delta / Alpha, Fp1Delta / Beta, Fp1 Delta / High Beta, Fp1 Theta / Alpha, … Pz Beta / High Beta
18399-19348	('abs_1_ec_FP1-LE', '1 Hz'), … ('abs_1_ec_Pz-LE', '50 Hz')	FFT Absolute Power 1 Hz	19 electrodes × [1-50] Hz, with this order: Fp1-1Hz, Fp1-2(Hz), Fp1-3(Hz), … Pz-49(Hz), Pz-50(Hz).
19349-19918	('z_abs_1_ec_FP1-LE', '1 Hz'), … ('z_abs_1_ec_Pz-LE', '30 Hz')	Z score Absolute Power 1 (Hz)	19 electrodes × [1-30] (Hz), with this order: Fp1-1(Hz), Fp1-2(Hz), Fp1-3(Hz), … Pz-29(Hz), Pz-30(Hz).
19919-20868	('rel_1_ec_FP1-LE', '1 Hz'), … ('rel_1_ec_Pz-LE', '50 Hz')	FFT Ralative Power 1 (Hz)	19 electrodes × [1-50] (Hz), with this order: Fp1-1(Hz), Fp1-2(Hz), Fp1-3(Hz), … Pz-49(Hz), Pz-50(Hz).
20869-21438	('z_rel_1_ec_FP1-LE', '1 Hz'), … ('z_rel_1_ec_Pz-LE', '30 Hz')	Z score Relative Power 1 (Hz)	19 electrodes × [1-30] (Hz), with this order: Fp1-1(Hz), Fp1-2(Hz), Fp1-3(Hz), … Pz-29(Hz), Pz-30(Hz).
21439-21704	('peak_ec_FP1-LE', 'Delta'), … ('peak_ec_Pz-LE', 'Gamma 2')	FFT Peak frequency	19 electrode × 14 Frequency Band, with this order: Fp1Delta, Fp1Theta, Fp1Alpha, … PzBeta3, PzGamma1, PzGamma2
21705-21894	('z_peak_ec_FP1-LE', 'Delta'), … ('z_peak_ec_Pz-LE', 'Beta 3')	Z score Peak frequency	19 electrode × 10 Frequency Band, with this order: Fp1Delta, Fp1Theta, Fp1Alpha, … PzBeta3
21895-24288	('amp_ec_FP1 FP2', 'Delta'), … ('amp_ec_Cz Pz', 'Gamma 2')	FFT Amplitude Asymmetry	((19 Electrodes × 18 Electrodes)/2) × 14 Frequency Band, with this order: FP1 FP2Delta, FP1 FP2Theta, FP1 FP2Alpha, … Cz PzGamma1, Cz PzGamma2
24289-25998	('z_amp_ec_FP1 FP2', 'Delta'), … ('z_amp_ec_Cz Pz', 'Beta 3')	Z score Amplitude Asymmetry	((19 Electrodes × 18 Electrodes)/2) × 10 Frequency Band, with this order: FP1 FP2Delta, FP1 FP2Theta, FP1 FP2Alpha, … Cz PzBeta2, Cz PzBeta3
25999-28392	('coh_ec_FP1 FP2', 'Delta'), … ('coh_ec_Cz Pz', 'Gamma 2')	FFT Coherence	((19 Electrodes × 18 Electrodes)/2) × 14 Frequency Band, with this order: FP1 FP2Delta, FP1 FP2Theta, FP1 FP2Alpha, … Cz PzGamma1, Cz PzGamma2
28393-30102	('z_coh_ec_FP1 FP2', 'Delta'), … ('z_coh_ec_Cz Pz', 'Beta 3')	Z score Coherence	((19 Electrodes × 18 Electrodes)/2) × 10 Frequency Band, with this order: FP1 FP2Delta, FP1 FP2Theta, FP1 FP2Alpha, … Cz PzBeta2, Cz PzBeta3
30103-32496	('phl_ec_FP1 FP2', 'Delta'), … ('phl_ec_Cz Pz', 'Gamma 2')	FFT Phase lag	((19 Electrodes × 18 Electrodes)/2) × 14 Frequency Band, with this order: FP1 FP2Delta, FP1 FP2Theta, FP1 FP2Alpha, … Cz PzGamma1, Cz PzGamma2
32497-34206	('z_phl_ec_FP1 FP2', 'Delta'), … ('z_phl_ec_Pz FP2', 'Beta3')	Z score Phase lag	((19 Electrodes × 18 Electrodes)/2) × 10 Frequency Band, with this order: FP1 FP2Delta, FP1 FP2Theta, FP1 FP2Alpha, … Cz PzBeta2, Cz PzBeta3

Table S2. Global and Special Analyses Quotient Scales provided by IVA-2

Column N° in Data file	Variable Name	Scale	Scale Description
34215	CACCQ	Accuracy	Number of times correct response is made (Combined)
34216	CCONQ	Consistency	Measure of ability to stay on task and sustain a reliable effort (Combined)
34217	CFOCQ	Focus	Total variability of mental processing speed for all correct responses during the test (Combined)
34218	CIAQ	Acuity	Measure of errors of omission under low demand conditions (Combined)
34219	CRELQ	Reliability	Measure of idiopathic errors of commission
34220	CSTDQ	Steadiness	Measure of accuracy in clicking to targets under high demand conditions (Combined)
34221	CMNQ	Speed	Average reaction time for all correct responses (Combined)
34222	CQUKQ	Quickness	Measure of response times when the targets are frequent (Combined)
34223	CMNLQ	Swiftness	Measure of response times when the targets are rare (Combined)
34224	CPOQ	Elasticity	Measure of the number of errors of omission occurring when a 1 is presented immediately after a 2 when the 1s are frequent (Combined)
34225	CPRQ	Prudence	Measure of impulsivity (Combined)
34226	CSTBQ	Stability	Variability of reaction time when targets are frequent (Combined)
34227	CSDLQ	Dependability	Variability of reaction times to targets under low demand conditions (Combined)
34228	CSTMQ	Stamina	Identifies difficulty in maintaining speed and sustaining attention and effort over time (Combined)
34229	CVIQ	Vigilance	Measure of inattention as evidenced by two different types of errors of omission (Combined)
34230	FRCQ	Response Control	Combination of Prudence, Consistency, and Stamina (Combined)
34231	ARCQ	Response Control	Combination of Prudence, Consistency, and Stamina (Auditory)
34232	VRCQ	Response Control	Combination of Prudence, Consistency, and Stamina (Visual)
34233	FAQ	Attention	Combination of Vigilance, Focus, and Speed (Combined)
34234	AAQ	Sustained Attention	Global measure of ability to respond to stimuli under low demand conditions accurately, quickly, and reliably (Auditory)
34235	VAQ	Sustained Attention	Global measure of ability to respond to stimuli under low demand conditions accurately, quickly, and reliably (Visual)
34236	SCQ	Self-Control	Combination of Prudence, Reliability, and Stamina (Combined)

34237	SCAQ	Self-Control	Combination of Prudence, Reliability, and Stamina (Auditory)
34238	SCVQ	Self-Control	Combination of Prudence, Reliability, and Stamina (Visual)
34239	PRQ	Prudence	Measure of impulsivity (Combined)
34240	PRAQ	Presence	Combination of Acuity, Elasticity, Steadiness, and Vigilance (Auditory)
34241	PRVQ	Presence	Combination of Acuity, Elasticity, Steadiness, and Vigilance (Visual)
34242	RSQ	Resilience	Combination of Consistency, Dependability, Focus, and Stability (Combined)
34243	RSAQ	Resilience	Combination of Consistency, Dependability, Focus, and Stability (Auditory)
34244	RSVQ	Resilience	Combination of Consistency, Dependability, Focus, and Stability (Visual)
34245	AGQ	Agility	Combination of Quickness, Speed, and Swiftness (Combined)
34246	AGAQ	Agility	Combination of Quickness, Speed, and Swiftness (Auditory)
34247	AGVQ	Agility	Combination of Quickness, Speed, and Swiftness (Visual)
34248	MCQ	Mental Concentration	Global measure of attentional functions (Combined)
34249	MCAQ	Mental Concentration	Global measure of attentional functions (Auditory)
34250	MCVQ	Mental Concentration	Global measure of attentional functions (Visual)
34251	CCOMQ	Competence	Measure of functioning under high demand conditions (Combined)
34252	COMAQ	Competence	Measure of functioning under high demand conditions (Auditory)
34253	COMVQ	Competence	Measure of functioning under high demand conditions (Visual)
34254	CSUSQ	Maintainability	Measure of functioning under low demand conditions (Combined)
34255	SUSAQ	Maintainability	Measure of functioning under low demand conditions (Auditory)
34256	SUSVQ	Maintainability	Measure of functioning under low demand conditions (Visual)
34257	SFAQ	Sustained Attention	Global measure of ability to respond to stimuli under low demand conditions accurately, quickly, and reliably (Combined)
34258	SAAQ	Sustained Attention	Global measure of ability to respond to stimuli under low demand conditions accurately, quickly, and reliably (Auditory)
34259	SVAQ	Sustained Attention	Global measure of ability to respond to stimuli under low demand conditions accurately, quickly, and reliably (Visual)

Table S3. Child Symptom Inventory–4 Scales

Column N° in Data file	Variable Name	Scale	Scale Description
34258-34275	A1 – A18	ADHD, Combine type	0 = never, 1 = sometimes, 2 = often, 3 = very often
34258-34266	A1 – A9	ADHD, Inattentive type	0 = never, 1 = sometimes, 2 = often, 3 = very often
34267-34275	A10 – A18	ADHD, Hyperactive type	0 = never, 1 = sometimes, 2 = often, 3 = very often
34276- 34283	B19 – B26	Oppositional defiant disorder	0 = never, 1 = sometimes, 2 = often, 3 = very often
34284- 34298	C27 – C41	Conduct disorder	0 = never, 1 = sometimes, 2 = often, 3 = very often
34259, 34301-34304, 34321	A2, D44 – D47, G64	Generalized anxiety disorder	0 = never, 1 = sometimes, 2 = often, 3 = very often
34306	E49	Specific phobia	0 = never, 1 = sometimes, 2 = often, 3 = very often
34307	E50	Obsession	0 = never, 1 = sometimes, 2 = often, 3 = very often
34308	E51	Compulsion	0 = never, 1 = sometimes, 2 = often, 3 = very often
34303	E52	Post-traumatic stress disorder	0 = never, 1 = sometimes, 2 = often, 3 = very often
34310	E53	Motor tic	0 = never, 1 = sometimes, 2 = often, 3 = very often
34311	E54	Vocal tic	0 = never, 1 = sometimes, 2 = often, 3 = very often
34312- 34316	F55 – F59	Schizophrenia	0 = never, 1 = sometimes, 2 = often, 3 = very often
34317- 34321 and 34325-34327	G60 – G64, G67 – G70	Major depressive disorder	0 = never, 1 = sometimes, 2 = often, 3 = very often
34302- 34317 and 34321-34325 and 343327	D45 – G60, G64 – G68, G70	Persistent depressive disorder	0 = never, 1 = sometimes, 2 = often, 3 = very often
34329-34340	H72 – H83	Autistic disorder	0 = never, 1 = sometimes, 2 = often, 3 = very often
34329- 34332 and 34337-34340	H72 – H75, H80 – H83	Asperger disorder	0 = never, 1 = sometimes, 2 = often, 3 = very often
34341- 34344	I84 – I87	Social phobia	0 = never, 1 = sometimes, 2 = often, 3 = very often
34345- 34352	J88 – J95	Separation anxiety disorder	0 = never, 1 = sometimes, 2 = often, 3 = very often
34353	J96	Enuresis	0 = never, 1 = sometimes, 2 = often, 3 = very often
34354	J97	Encopresis	0 = never, 1 = sometimes, 2 = often, 3 = very often

Table S4. Working Memory Task Scales

Column N° in Data file	Variable Name	Scale	Scale Description
34415-34426	('Hit Rate', 'Number', 'condition1') … ('Hit Rate', 'nonNumber', 'hit_rate_conds_negative_set')	Hit rate	Hit rate is calculated for each condition (4 negative set conditions and positive set condition) and for each block (block number and non-number), separately.
34427-34438	('False Alarm Rate', 'Number', 'condition1') … ('False Alarm Rate', 'nonNumber', 'false_rate_conds_negative_set')	False alarm rate	False alarm rate is calculated for each condition (4 negative set conditions and positive set condition) and for each block (block number and non-number), separately.
34439- 34450	('dPrime', 'Number', 'condition1') … ('dPrime', 'nonNumber', 'conds_negative_set(1-4)')	D-prime conditions	d' for each block and for each condition, separately.
34451, 34452	('dPrime', 'total', 'Number'), ('dPrime', 'total', 'nonNumber')	D-prime	Total d' for each block = $d'\ (block)\ = (d'(negative\ set)\ +\ d'\ (positive\ set))\ /\ 2$
34453	('Memory', 'Memory', 'value')	Grand total memory score	$d'\ (total)\ = (d'(block\ 1)\ +\ d'\ (block\ 2))\ /\ 2$

Table S5. Ravan Test Scales

Column N° in Data file	Variable Name	Scale	Scale Description
34454	raven	Raw Score	Total number of correct answers on the test
34455	iq	IQ Score	Converted score based on normative data (average = 100)
34456	percentage_rank	Percentage Rank	Indicates performance relative to peers (e.g., 75th percentile)
