# Supplementary material for: Iranian 6-11 years age population-based EEG, ERP, and cognition dataset
Source: Sci Data. 2025 Feb 22;12:319. doi: 10.1038/s41597-025-04624-6 (PMC11846862; doi:10.1038/s41597-025-04624-6)
Supplement: Supplementary file 2 — Supplementary Appendices [file 41597_2025_4624_MOESM2_ESM.pdf]

Supplementary Appendices

APPENDIX 1 : CHILD SYMPTOM INVENTORY-4 (CSI-4): PARENT CHECKLIST

APPENDIX 2: Edinburgh Handedness Inventory

APPENDIX 3 : Adult Reading History Questionnaire

APPENDIX 4 : Adult Arithmetic History Questionnaire

APPENDIX 1

CHILD SYMPTOM INVENTORY-4 (CSI-4): PARENT CHECKLIST

DIRECTIONS: CHECK WHICH RATING BEST DESCRIBES YOUR CHILD'S OVERALL BEHAVIOR. ANSWER EACH QUESTION TO THE BEST OF YOUR ABILITY.

	CATEGORY A:	NEVER	SOME-TIMES	OFTEN	VERY OFTEN
1	Fails to give close attention to details or makes careless mistakes				
2	Has difficulty paying attention to tasks or play activities				
3	Does not seem to listen when spoken to directly				
4	Has difficulty following through on instructions and fails to finish things				
5	Has difficulty organizing tasks and activities				
6	Avoids doing tasks that require a lot of mental effort (schoolwork, homework, etc.)				
7	Loses things necessary for activities				
8	Is easily distracted by other things going on				
9	Is forgetful in daily activities				
10	Fidgets with hands or feet or squirms in seat				
11	Has difficulty remaining seated when asked to do so				
12	Runs about or climbs on things when asked not to do so				
13	Has difficulty playing quietly				
14	Is "on the go" or acts as if "driven by a motor"				
15	Talks excessively				
16	Blurts out answers to questions before they have been completed				
17	Has difficulty awaiting turn in group activities				
18	Interrupts people butts into other children's activities				

	CATEGORY B:	NEVER	SOME-TIMES	OFTEN	VERY OFTEN
19	Loses temper				
20	Argues with adults				
21	Defies or refuses what you tell him/her to do				
22	Does things to deliberately annoy others				
23	Blames others for own misbehavior or mistake				
24	Is touchy or easily annoyed by others				
25	Is angry and resentful				
26	Takes anger out on others or tries to get even				

	CATEGORY C:	NEVER	SOME-TIMES	OFTEN	VERY OFTEN
27	Plays hookey from school				
28	Stays out at night when not supposed to				
29	Lies to get things or to avoid responsibility ("cons" others)				
30	Bullies, threatens, or intimidates others				
31	Starts physical fights				
32	Has run away from home overnight				
33	Has stolen things when others were not looking				
34	Has deliberately destroyed others' property				
35	Has deliberately started fires				
36	Has stolen things from others using physical force				
37	Has broken into someone else's house, building, or car				
38	Has used a weapon when fighting (bat, brick bottle, etc.)				
39	Has been physically cruel to animals				
40	Has been physically cruel to people				
41	Has been preoccupied with or involved in sexual activity				

	CATEGORY D:	NEVER	SOME-TIMES	OFTEN	VERY OFTEN
42	Is overconcerned about abilities in academic, athletic, or social activities				
43	Has difficulty controlling worries				
44	Acts restless or edgy				
45	Is irritable for most of the day				
46	Is extremely tense or unable to relax				
47	Has difficulty falling asleep or staying asleep				
48	Complains about physical problems (headaches, upset stomach, etc.) for which there is no apparent cause				

	CATEGORY E:	NEVER	SOME-TIMES	OFTEN	VERY OFTEN
49	Shows excessive fear to specific objects or situations (animals, heights, storms, insects, etc.)				
50	Can not get distressing thoughts out of his/her mind (worries about germs or doing things perfectly, etc.)				
51	Feels compelled to perform unusual habits (hand washing, checking locks, repeating things a set number of times)				
52	Has experienced an extremely upsetting event and continues to be bothered by it				
53	Does unusual movements for no apparent reason (eye blinking, twitching, lip licking, head jerking, etc.)				
54	Makes vocal sounds for no apparent reason (coughing, throat clearing, sniffling, grunting, etc.)				

	CATEGORY F:	NEVER	SOME-TIMES	OFTEN	VERY OFTEN
55	Has strange ideas or beliefs that are not real (child's food is poisoned, people are trying to get him/her, etc.)				
56	Has auditory hallucinations-hears voices talking to or telling him/her to do things				
57	Has extremely strange and illogical thoughts or ideas				
58	Laughs or cries at inappropriate times or shows no emotion in situations where most others of same age would react				
59	Does extremely odd things (excessive preoccupation with fantasy friends, talks to self in a strange way, etc.)				

	CATEGORY G:	NEVER	SOME-TIMES	OFTEN	VERY OFTEN
60	Is depressed for most of the day				
61	Shows little interest in (or enjoyment of) pleasurable activities				
62	Has recurrent thoughts of death or suicide				
63	Feels worthless or guilty				
64	Has low energy level or is tired for no apparent reason				
65	Has little confidence or is very self-conscious				
66	Feels that things never work out right				

.

67	Has experienced a big change in his/her normal appetite or weight (circle yes or no)	NO	YES
68	Has experienced a big change in his/her normal sleeping habits--cannot sleep or sleeps too much (circle yes or no)	NO	YES
69	Has experienced a big change in his/her normal activity level--overactive or inactive (circle yes or no)	NO	YES
70	Has experienced a big change in his/her ability to concentrate (circle yes or no)	NO	YES
71	Has experienced a big drop in school grades or schoolwork (circle yes or no)	NO	YES

	CATEGORY H:	NEVER	SOME-TIMES	OFTEN	VERY OFTEN
72	Has a peculiar way of relating to others (avoids eye contact, odd facial expressions or gestures, etc.)				
73	Does not play or relate well with other children				
74	Not interested in making friends				
75	Is unaware or takes no interest in other people's feeling				
76	Has a significant problem with language				
77	Has difficulty making socially appropriate conversation				
78	Talks in a strange way (repeats what others say; confuses words like “you” and “I” uses odd words or phrases, etc.)				
79	Is unable to “pretend” or “make believe” when playing				
80	Shows excessive preoccupation with one topic				
81	Gets very upset over small changes in routine or surroundings				
82	Makes strange repetitive movements (flapping arms, etc.)				
83	Has strange fascination for parts of objects				

	CATEGORY I:	NEVER	SOME-TIMES	OFTEN	VERY OFTEN
84	Tries to avoid contact with strangers; abnormally shy				
85	Is excessively shy with peers				
86	Is generally warm and outgoing with family members and familiar adults				
87	When put in an uncomfortable social situation, child cries, freezes, or withdraws from interacting				

	CATEGORY J:	NEVER	SOME-TIMES	OFTEN	VERY OFTEN
88	Gets very upset when child expects to be separated from home or parents				
89	Worries that parents will be hurt or leave home and not come back				
90	Worries that some disaster (getting lost, kidnapped, etc.) will separate child from parents				
91	Tries to avoid going to school in order to stay home with parent				
92	Worries about being left at home alone or with a sitter				
93	Afraid to go to sleep unless near parent				
94	Has nightmares about being separated from parent				
95	Complains about feeling sick when child expects to be separated from home or parents				
96	Wets bed at night				
97	Wets or soils underwear during daytime hours				

APPENDIX 2

Edinburgh Handedness Inventory

WHICH HAND DOES YOUR CHILD USE MOST TO DO THE FOLLOWING TASKS?

	Always Left	Usually Left	No Preference	Usually Right	Always Right
1. Writing					
2. Drawing					
3. Throwing					
4. Scissors					
5. Toothbrush					
6. Knife (without fork)					
7. Spoon					
8. Broom (Upper hand)					
9. Striking match (match)					
10. Opening box/lid					

	Not at all 0	1	2	3	Very much 4
11. Does your child have a problem distinguishing his/her left and right?					
12. Does your child have a problem with navigation or finding an address?					

APPENDIX 3
Adult Reading History Questionnaire

PLEASE NOTE: This reading history questionnaire applies to you, and not to your children.

Please circle the number of the response that most nearly describes your attitude or experience for each of the following questions or statements. If you think your response would be between numbers, place an "X" where you think it should be.

1. Which of the following most nearly describes your attitude toward school when you were a child:

**Loved school; favorite activity**				**Hated school; tried to get out of going**
**0 _______**	**_______ 1 _______**	**_______ 2 _______**	**_______ 3 _______**	**_______ 4**

2. How much difficulty did you have learning to read in elementary school?

**None**				**A great deal**
**0 _______**	**_______ 1 _______**	**_______ 2 _______**	**_______ 3 _______**	**_______ 4**

3. How much extra help did you need when learning to read in elementary school?

**Help from: No help**	**Friends**	**Teachers/ parents**	**Tutors or special class 1 year**	**Tutors or special class 2 or more years**
**0 _______**	**_______ 1 _______**	**_______ 2 _______**	**_______ 3 _______**	**_______ 4**

4. Did you ever reverse the order of letters or numbers when you were a child?

**No**				**A great deal**
**0 _______**	**_______ 1 _______**	**_______ 2 _______**	**_______ 3 _______**	**_______ 4**

5. Did you have difficulty learning letter and/or color names when you were a child?

**No**				**A great deal**
**0 _______**	**_______ 1 _______**	**_______ 2 _______**	**_______ 3 _______**	**_______ 4**

6. How would you compare your reading skill to that of others in your elementary classes?

**Above average**		**Average**		**Below average**
**0 _______**	**_______ 1 _______**	**_______ 2 _______**	**_______ 3 _______**	**_______ 4**

7. All students struggle from time to time in school. In comparison to others in your classes, how much did you struggle to complete your work?

**Not at all**	**Less than most**	**About the same**	**More than most**	**Much more than most**
**0 _______**	**_______ 1 _______**	**_______ 2 _______**	**_______ 3 _______**	**_______ 4**

8. Did you experience difficulty in high school or college English* classes?

**No; enjoyed and did well**		**Some**		**A great deal; did poorly**
**0 _______**	**_______ 1 _______**	**_______ 2 _______**	**_______ 3 _______**	**_______ 4**

9. What is your current attitude toward reading?

**Very positive**				**Very negative**
**0 _______**	**_______ 1 _______**	**_______ 2 _______**	**_______ 3 _______**	**_______ 4**

10. How much reading do you do for pleasure?

**A great deal**		**Some**		**None**
**0 _______**	**_______ 1 _______**	**_______ 2 _______**	**_______ 3 _______**	**_______ 4**

11. How would you compare your current reading speed to that of others of the same age and education?

**Above average**		**Average**		**Below average**
**0 _______**	**_______ 1 _______**	**_______ 2 _______**	**_______ 3 _______**	**_______ 4**

12. How much reading do you do in conjunction with your work? (If retired or not working, how much did you read when you were working?)

**A great deal**		**Some**		**None**
**0 _______**	**_______ 1 _______**	**_______ 2 _______**	**_______ 3 _______**	**_______ 4**

13. How much difficulty did you have learning to spell in elementary school?

**None**		**Some**		**A great deal**
**0 _______**	**_______ 1 _______**	**_______ 2 _______**	**_______ 3 _______**	**_______ 4**

14. How would you compare your current spelling to that of others of the same age and education?

**Above average**		**Average**		**Below average**
**0 _______**	**_______ 1 _______**	**_______ 2 _______**	**_______ 3 _______**	**_______ 4**

15. Did your parents ever consider having you repeat any grades in school due to academic failure (not illness)?

**No**	**Talked about it, but didn't do it**	**Repeated 1 grade**	**Repeated 2 grades**	**Dropped out**
**0 _______**	**_______ 1 _______**	**_______ 2 _______**	**_______ 3 _______**	**_______ 4**

16. Do you ever have difficulty remembering people's names or names of places?

**No**				**A great deal**
**0 _______**	**_______ 1 _______**	**_______ 2 _______**	**_______ 3 _______**	**_______ 4**

17. Do you have difficulty remembering addresses, phone numbers, or dates?

**No**				**A great deal**
**0 _______**	**_______ 1 _______**	**_______ 2 _______**	**_______ 3 _______**	**_______ 4**

18. Do you have difficulty remembering complex verbal instructions?

**No**				**A great deal**
**0 _______**	**_______ 1 _______**	**_______ 2 _______**	**_______ 3 _______**	**_______ 4**

19. Do you currently reverse the order of letters or numbers when you read or write?

**No**				**A great deal**
**0 _______**	**_______ 1 _______**	**_______ 2 _______**	**_______ 3 _______**	**_______ 4**

20. How many books do you read for pleasure each year?

**More than 10**	**6-10**	**2-5**	**1-2**	**None**
**0 _______**	**_______ 1 _______**	**_______ 2 _______**	**_______ 3 _______**	**_______ 4**

21. How many magazines do you read for pleasure each month?

**5 or more**	**3-4 regularly**	**1-2 regularly**	**1-2 irregularly**	**None**
**0 _______**	**_______ 1 _______**	**_______ 2 _______**	**_______ 3 _______**	**_______ 4**

22. Do you read daily (Monday-Friday) newspapers?

**Every day**	**Once a week**	**Once in a while**	**Rarely**	**Never**
**0 _______**	**_______ 1 _______**	**_______ 2 _______**	**_______ 3 _______**	**_______ 4**

23. Do you read a newspaper on Sunday*?

**Completely every Sunday**	**Scan each week**	**Once in a while**	**Rarely**	**Never**
**0 _______**	**_______ 1 _______**	**_______ 2 _______**	**_______ 3 _______**	**_______ 4**

*****Check the most appropriate answer for each of the following questions*****

24. To the best of your knowledge, did your parents ever report that either one of them had a problem with reading or spelling?

______________Yes

______________No

______________Not sure

If yes, please give details:

25. To the best of your knowledge, did your brothers and/or sisters ever have a problem with reading or spelling?

______________Yes

______________No

______________Not sure

If yes, please give details:

* In the Persian version of the questionnaire, for cultural adaptation, in question 8, the “English” word was replaced with “Farsi”, and in question 23, the word “Sunday” was replaced with “Friday”.

APPENDIX 4

Adult Arithmetic History Questionnaire

PLEASE NOTE: This reading history questionnaire applies to you, and not to your children.

Please circle the number of the response that most nearly describes your attitude or experience for each of the following questions or statements. If you think your response would be between numbers, place an "X" where you think it should be.

1. Which of the following most nearly describes your attitude toward arithmetic when you were a child?

Very positive				Very negative
0 _______	_______ 1 _______	_______ 2 _______	_______ 3 _______	_______ 4

2. How much difficulty did you have learning math facts (addition, subtraction, multiplication, division) in elementary school?

None				A great deal
0 _______	_______ 1 _______	_______ 2 _______	_______ 3 _______	_______ 4

3. How much extra help did you need when learning math in elementary school?

No help	Help from friends	Help from teachers/ parents	Tutors or special class 1 year	Tutors or special class 2+ years
0 _______	_______ 1 _______	_______ 2 _______	_______ 3 _______	_______ 4

4. Did you have difficulty learning number names and counting when you were a child?

No				A great deal
0 _______	_______ 1 _______	_______ 2 _______	_______ 3 _______	_______ 4

5. Did you have poor memory for numbers when you were a child?

No				A great deal
0 _______	_______ 1 _______	_______ 2 _______	_______ 3 _______	_______ 4

6. Did you ever reverse the order of numbers or counted incorrectly when you were a child?

No				A great deal
0 _______	_______ 1 _______	_______ 2 _______	_______ 3 _______	_______ 4

7. How would you compare your math skill to that of others in your elementary classes?

Above average		Average		Below average
0 _______	_______ 1 _______	_______ 2 _______	_______ 3 _______	_______ 4

8. Did you have difficulty developing math problem-solving skills in elementary school?

No				A great deal
0 _______	_______ 1 _______	_______ 2 _______	_______ 3 _______	_______ 4

9. All students struggle with math from time to time in school. In comparison to others in your classes. How much did you struggle to complete your math work?

Not at all	Less than most	About the same	More than most	Much more than most
0 _______	_______ 1 _______	_______ 2 _______	_______ 3 _______	_______ 4

10. Do you experience difficulty in estimating costs such as groceries bills?

No				A great deal
0 _______	_______ 1 _______	_______ 2 _______	_______ 3 _______	_______ 4

11. What is your current attitude toward arithmetic?

Very positive				Very negative
0 _______	_______ 1 _______	_______ 2 _______	_______ 3 _______	_______ 4

12. Do you use mental arithmetic to cope with every-day calculations ordo you prefer to use a calculator (e.g., during shopping or paying bills)?

Mostly use mental math		Use mental math and calculators equally		Mostly use calculators
0 _______	_______ 1 _______	_______ 2 _______	_______ 3 _______	_______ 4

13. How would you compare your current arithmetical skills to that of others of the same age and education?

Above average		Average		Below average
0 _______	_______ 1 _______	_______ 2 _______	_______ 3 _______	_______ 4

14. Do you struggle with understanding concepts and estimating time and/or distance?

No				A great deal
0 _______	_______ 1 _______	_______ 2 _______	_______ 3 _______	_______ 4

15. How much difficulty did you have learning the multiplication table in elementary school?

None		Some		A great deal
0 _______	_______ 1 _______	_______ 2 _______	_______ 3 _______	_______ 4

16. Do you have difficulty remembering addresses, phone numbers, or dates?

None		Some		A great deal
0 _______	_______ 1 _______	_______ 2 _______	_______ 3 _______	_______ 4

17. Do you have trouble remembering numbers or instruction that involve numbers and simple calculation (e.g., prices, percentage, medication dosage)?

No				A great deal
0 _______	_______ 1 _______	_______ 2 _______	_______ 3 _______	_______ 4

18. Do you currently reverse the order of numbers when you read or write numbers, or do math?

None				A great deal
0 _______	_______ 1 _______	_______ 2 _______	_______ 3 _______	_______ 4

19. Did you avoid using mathematics during your education?

None				A great deal
0 _______	_______ 1 _______	_______ 2 _______	_______ 3 _______	_______ 4

20. Would you prefer to write an essay than to do an assignment in math?

Prefer to do math		No preference		Prefer to write an essay
0 _______	_______ 1 _______	_______ 2 _______	_______ 3 _______	_______ 4

21. How much do you use numbers to describe measures such as distance or weight?

Every day	Once a week	Once in a while	Rarely	Never
0 _______	_______ 1 _______	_______ 2 _______	_______ 3 _______	_______ 4

22. Do you struggle with understanding basic arithmetical concepts such as the base-10 system and calculating with decimals and fractions?

None				A great deal
0 _______	_______ 1 _______	_______ 2 _______	_______ 3 _______	_______ 4

23. Do you like doing games that involve numbers (e.g., such as soduko or monopoly) in your free time?

Every day	Once a week	Once in a while	Rarely	Never
0 _______	_______ 1 _______	_______ 2 _______	_______ 3 _______	_______ 4
